# Loss of PTEN stabilizes the lipid modifying enzyme cytosolic phospholipase A_2_α *via* AKT in prostate cancer cells

**DOI:** 10.18632/oncotarget.2198

**Published:** 2014-07-09

**Authors:** Soma Vignarajan, Chanlu Xie, Mu Yao, Yuting Sun, Ulla Simanainen, Paul Sved, Tao Liu, Qihan Dong

**Affiliations:** ^1^ Discipline of Endocrinology, Central Clinical School, Bosch Institute, Royal Prince Alfred Hospital, and Charles Perkins Centre, The University of Sydney, Sydney, NSW, Australia; ^2^ Children's Cancer Institute Australia for Medical Research, Sydney, Australia; ^3^ ANZAC Research institute, The University of Sydney, Sydney, NSW, Australia; ^4^ Sydney Cancer Centre, Royal Prince Alfred Hospital, Camperdown, NSW, Australia; ^5^ School of Women's and Children's Health, UNSW Medicine, Sydney, Australia, Australia; ^6^ School of Science and Health, The University of Western Sydney, Sydney, Australia

**Keywords:** cytosolic phospholipase A2, AKT, PTEN, prostate cancer

## Abstract

Aberrant increase in pAKT, due to a gain-of-function mutation of *PI3K* or loss-of-function mutation or deletion of *PTEN,* occurs in prostate cancer and is associated with poor patient prognosis. Cytosolic phospholipase A_2_α (cPLA_2_α) is a lipid modifying enzyme by catalyzing the hydrolysis of membrane arachidonic acid. Arachidonic acid and its metabolites contribute to survival and proliferation of prostate cancer cells. We examined whether AKT plays a role in promoting cPLA_2_α action in prostate cancer cells. We found a concordant increase in pAKT and cPLA_2_α levels in prostate tissue of prostate epithelial-specific *PTEN*-knockout but not *PTEN*-wide type mice. Restoration of PTEN expression or inhibition of PI3K action decreased cPLA_2_α expression in *PTEN*-mutated or deleted prostate cancer cells. An increase in AKT by Myr-AKT elevated cPLA_2_α protein levels, which could be diminished by inhibition of AKT phosphorylation without noticeable change in total AKT levels. pAKT levels had no influence on cPLA_2_α at mRNA levels but reduced cPLA_2_α protein degradation. Anti-AKT antibody co-immunoprecipitated cPLA_2_α and vice versa. Hence, AKT plays a role in enhancing cPLA_2_α protein stability in PTEN-null prostate cancer cells, revealing a link between oncogenic pathway and lipid metabolism.

## INTRODUCTION

Membrane phosphatidylinositol-(3,4,5)-trisphosphate (PIP3) provides the anchor site for PH-domain-containing proteins including protein kinase B (AKT) [1]. Due to close proximity to PH-domain-containing kinases, AKT is activated by phosphorylation on cell membrane [2]. The phosphorylated AKT (pAKT) then phosphorylates a variety of target proteins, leading to an increase in cell survival and proliferation [2]. The homeostasis of PIP3 levels are maintained through phosphoinositide-3-kinase (PI3K) and phosphatase and tensin homolog (PTEN). PI3K phosphorylates phosphatidylinositol-(4,5)-diphosphate (PIP2) to generate PIP3, whereas PTEN dephosphorylates PIP3 to generate PIP2 [3]. Thus, PI3K and PTEN are positive and negative regulators of pAKT, respectively [3]. Genetic alterations of *PI3K* and *PTEN* have been found in at least sixteen types of human cancers [4]. Nearly 30-60% prostate cancer cases have either gain-of-function-mutation in *PI3K* or loss-of-function-mutation or deletion in *PTEN* [4]. About 45% of prostate cancer cases have increased levels of pAKT, which correlates with the disease severity [5, 6]. The loss of PTEN or increase in pAKT at Ser^473^ has been used to predict advanced prostate cancer that will fail to respond to treatment [7-9].

Studies have shown that polyunsaturated fatty acid, *e.g.* arachidonic acid (AA), promotes prostate cancer progression. High dietary AA reduces the time required to convert hormone sensitive to refractory prostate cancer [10]. Mice supplemented with AA in the diet show earlier, more frequent and larger tumor recurrence than controls following the surgical removal of prostate cancer xenograft, which imitates prostatectomy in clinical setting [11]. Dietary AA enhances tumor growth in prostate-specific PTEN-knockout mice [12]. *In vitro*, AA [13] and its metabolites (i.e., eicosanoids) [14] stimulate the survival and proliferation of human prostate cancer cells. The rate of eicosanoid production is also significantly higher in malignant prostate cancer tissues than benign prostate tissues [15]. In two clinical trials, the rate of increase in prostate specific antigen (PSA) following prostatectomy or radiotherapy was slowed in patients treated with inhibitors of eicosanoid-producing enzymes [16, 17]. Collectively, these studies demonstrate a role of AA and its metabolites in prostate cancer progression.

As a derivative of essential fatty acid linoleic acid, the AA is taken up at the cell membrane and esterified mainly into the *sn*-2 position of glycerophospholipid [18]. The biological action of AA begins after its hydrolysis by phospholipase A_2_ (PLA_2_). Of the PLA_2_ family, the cytosolic PLA_2_α (cPLA_2_α) is the isoform that cleaves AA specifically at the *sn*-2 position [19]. We [14, 20-23] and others [24-26] have shown that cPLA_2_α action contributes to survival, proliferation, or motility of prostate cancer cells. Blocking cPLA_2_α with a gene silencing or pharmacological approach retards proliferation of prostate cancer cells *in vitro* or *in vivo* [23].

Since both pAKT and cPLA_2_α levels are implicated in the prostate cancer, and an understanding of the integration of biochemical pathways involved in cancer progression is a key to the development of improved pharmacological treatment strategies for cancer [27, 28], we aimed to examine the relationship between the oncogenic protein and the lipid modifying enzyme. Specifically, we verified the concordance between pAKT and cPLA_2_α in prostate tissue of epithelial-specific *PTEN*-knockout mouse, and tested the hypothesis that pAKT plays a causal role in promoting cPLA_2_α expression in prostate cancer cells.

## RESULTS

### Concordant change between pAKT and cPLA_2_α levels in PTEN-knockout mouse prostate

**Figure 1:**
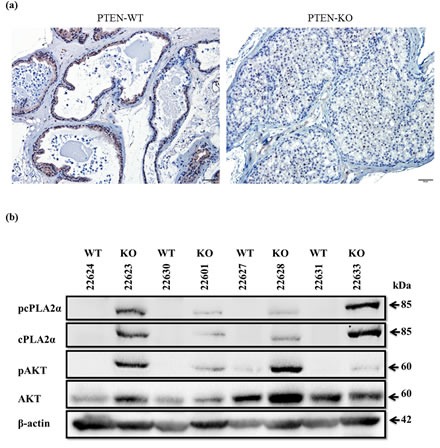
PTEN, AKT and cPLA_2_α in the prostate of mouse with prostate-epithelial specific knockout (KO) and wide type (WT) of *PTEN* (a): Prostate tissue of 6 week-old *PTEN* KO and WT mice were dissected, fixed, and processed for paraffin blocks. Sections were cut for immunostaining of PTEN. Scale bar: 50 μm. (b): Fresh frozen prostate tissue of 6 week-old *PTEN* KO and WT were homogenized. The resultant supernatants were used for immunoblot of AKT and cPLA_2_α.

To determine the relationship between pAKT and cPLA_2_α *in vivo*, we firstly confirmed the PTEN status by immunostaining of prostate tissues collected from prostate-epithelial-specific *PTEN*-KO and *PTEN*-WT mice (Figure 1a). While PTEN was present in both epithelial and stromal compartments of *PTEN*-WT mice, this trait was absent in cancer in *PTEN*-KO mice. However, PTEN remained in stromal cells in the *PTEN*-KO, confirming a selective deletion of PTEN in prostate epithelium. To determine the relationship between pAKT and cPLA_2_α levels, we determined by immunoblot the AKT and cPLA_2_α protein levels in the tissue. While the levels of total AKT were not altered significantly, there was a clear increase in pAKT at Ser^473^ in *PTEN*-KO compared to *PTEN*-WT mice (Figure 1b). Interestingly, the levels of total cPLA_2_α and pcPLA_2_α at Ser^505^ were increased significantly in *PTEN*-KO mice. Hence, pAKT levels are positively correlated with cPLA_2_α in rodent prostate cancer induced by *PTEN*-KO.

### Decrease in pAKT reduces cPLA_2_α expression and phosphorylation in prostate cancer cells

**Figure 2:**
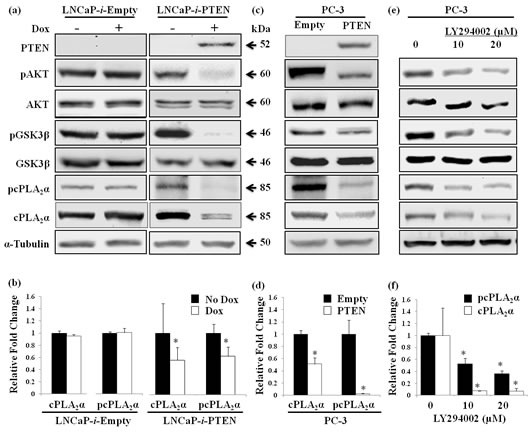
Effect of PTEN expression or PI3K inhibition on cPLA_2_α protein levels (a) LNCaP cells carrying *tet* repressor were stably transfected with either Dox-inducible PTEN (LNCaP-*i*-PTEN) or the empty vector (LNCaP-*i*-Empty), and treated with or without Dox (100 ng/mL) for 24 h. The harvested cells were lysed and supernatants were used for immunoblot. (b) Quantified band intensity of 3 independent experiments is represented as the mean ± SD. * P<0.05 versus no Dox treatment. (c) PC-3 cells stably transfected with *PTEN* or empty vector were lysed for immunoblot. (d) Quantified band intensity of 3 independent experiments is represented as the mean ± SD. * P<0.05 versus empty vector-transfected cells. (e) PC-3 cells treated with PI3K inhibitor (LY294002) for 1 h at indicated doses were harvested at 24 h for immunoblot. (f) Quantified band intensity of 3 independent experiments is represented as the mean ± SD. * P<0.05 versus vehicle-treated control.

To determine if a causal relationship exists between pAKT and cPLA_2_α, we established a Dox-controlled *PTEN* expression system in LNCaP cells, which has a frame-shift mutation in *PTEN* gene resulting in a truncated non-functional PTEN protein [29]. Dox-induced *PTEN* expression caused a significant decrease in pAKT at Ser^473^. Concomitantly, phosphorylation of its immediate downstream target GSK3β at Ser^9^ (Figure 2a) was also diminished. In contrast, total AKT and GSK3β remained unchanged. Control cells transfected with same vector but without *PTEN* sequence showed no change in pAKT and pGSK3β following Dox treatment. Interestingly, the decrease in pAKT by restoration of *PTEN* caused reduction of the levels of total cPLA_2_α and phospho-cPLA_2_α (pcPLA_2_α) at Ser^505^ (Figure 2a, b). Due to the change in configuration following phosphorylation at Ser^505^, pcPLA_2_α enhances AA releasing property [30]. In control cells, there was no change in cPLA_2_α expression or phosphorylation following Dox treatment (Figure 2a, b). As expected, PTEN restoration also reduced the proliferation and increased apoptosis in LNCaP cells compared with control cells which had no functional PTEN (Supplemental Figure 1).

To confirm the effect of PTEN restoration on cPLA_2_α, we stably transfected another prostate cancer cell line PC-3 with a *PTEN*-expression construct. PC-3 has a deletion in *PTEN* gene and thus has no PTEN proteins [31]. Ectopic expression of PTEN caused the reduction of pAKT at Ser^473^and pGSK3β at Ser^9^ in PC-3 cells in the absence of alterations in total AKT and GSK3β (Figure 2d). Again, there was a significant decrease in cPLA_2_α and pcPLA_2_α at Ser^505^ in *PTEN* compared with empty vector transfected PC-3 cells (Figure 2c, d). As expected, PC-3 cell proliferation was reduced after PTEN restoration.

To verify if the regulation of cPLA_2_α by PTEN is *via* pAKT, we blocked PI3K enzyme action with LY294002 in PC-3 cells. Indeed, blocking PI3K led to a decrease in levels of pAKT at Ser^473^ and pGSK3β at Ser^9^, while there was no change in total AKT and GSK3β (Figure 2e). Similarly, total cPLA_2_α and pcPLA_2_α at Ser^505^ levels were decreased in PC-3 cells treated with PI3K inhibitor compared with vehicle-treated control cells (Figure 2e, f). Taken together, manipulation of pAKT positive regulator (PI3K) or negative regulator (PTEN) changes cPLA_2_α expression and phosphorylation; suggesting a role of pAKT in the regulation of cPLA_2_α in prostate cancer cells.

### Increase in pAKT elevates cPLA_2_α expression in prostate cancer cells

**Figure 3:**
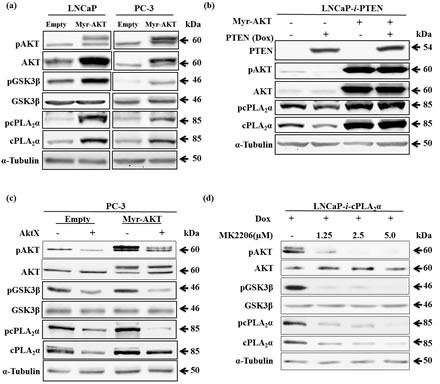
Effect of Myr-AKT expression on cPLA_2_α protein levels in the presence or absence of PTEN (a) LNCaP or PC-3 cells transiently transfected with Myr-AKT or empty vector (2 μg, 24 h) were harvested 24 h later. The cell lysates were used for immunoblot. (b) LNCaP-*i*-PTEN cells transiently transfected with Myr-AKT or empty vector (2 μg, 24 h) were treated with or without Dox (100 ng/mL) for another 24 h. The cell lysates were used for immunoblot. (c) PC-3 cells were transfected with Myr-AKT (2 μg, 24 h) followed by incubation with the AKT phosphorylation inhibitor AktX (5 μM, 1 h). The cells were harvested 24 h later. (d) LNCaP cells stably transfected with Dox-inducible cPLA_2_α expression system (LNCaP-*i*-cPLA_2_α) were treated with Dox (100 ng/mL, 24 h) and then incubated with AKT phosphorylation inhibitor MK2206 at indicated doses for 1 h. The cells were harvested 24 h later. All results are typical of 3 independent experiments.

To determine the effect of an increase in pAKT levels on total cPLA_2_α and pcPLA_2_α levels, we transiently transfected LNCaP and PC-3 cells with an expression vector containing *Myr-AKT*. Due to the addition of the sequence coding for myristoylation signal in *AKT* construct, the produced AKT protein is able to bind to membrane independent from PIP3 and being phosphorylated. The transfection with *Myr-AKT* in both LNCaP and PC-3 cells caused an increase in total AKT, pAKT and pGSK3β (Figure 3a). Concomitantly, there was a clear increase in total cPLA_2_α and pcPLA_2_α (Figure 3a). Then, we determined the effect of increase in pAKT on cPLA_2_α in PTEN-positive prostate cancer cells. Myr-AKT was transiently transfected into LNCaP cells in which PTEN had been restored. In the presence of Myr-AKT, the inhibitory effect of PTEN restoration on cPLA_2_α expression and phosphorylation was abolished (Figure 3b).

To further validate the importance of pAKT on cPLA_2_α expression and phosphorylation, we introduced AKT inhibitor, AktX, in the presence or absence of Myr-AKT in PC-3 cells. Indeed, blocking AKT phosphorylation with AktX significantly decreased total cPLA_2_α and pcPLA_2_α while total AKT was unchanged (Figure 3c). We then confirmed the finding in LNCaP cells with another AKT inhibitor (MK2206). As endogenous cPLA_2_α levels were relatively low in LNCaP cells, we established a Dox-controlled *cPLA_2_α* expression system in LNCaP cells and then introduced MK2206 in the presence of Dox. Blocking AKT phosphorylation with MK2206 significantly decreased total cPLA_2_α and pcPLA_2_α while total AKT was unchanged (Figure 3c). These results confirm that phosphorylated form of AKT is required for the effect on cPLA_2_α protein levels in prostate cancer cells.

### Regulation of cPLA_2_α by pAKT is independent of extracellular signal-regulated kinase (ERK^1/2^)

**Figure 4:**
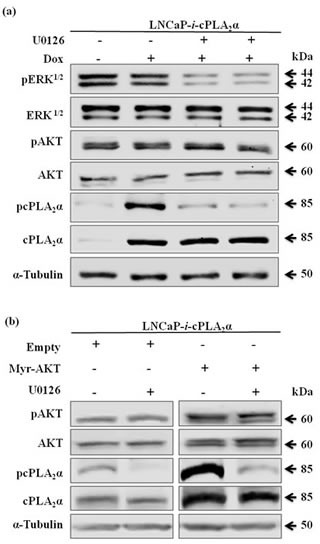
Effect of pAKT on cPLA_2_α is independent of ERK^1/2^ (a) LNCaP cells stably transfected with Dox-controlled inducible cPLA_2_α expression system (LNCaP-*i*-cPLA_2_α) were treated with Dox (100 ng/mL, 24 h), followed by incubation with MEK inhibitor U0126 (5 μM, 1 h). The cells were harvested 24 h later. (b) LNCaP-*i-*cPLA_2_α cells induced with Dox for 1 h and transfected with Myr-AKT or empty vector (2 μg, 24 h), followed by incubation with U0126 (5 μM, 1 h). The cells were harvested 24 h later. All immunoblotting results are typical of 3 independent experiments.

Previous studies have shown a regulation of cPLA_2_α phosphorylation at ser^505^ by mitogen-activated protein (MAP) kinase ERK^1/2^ [32-34]. To determine if the regulation of cPLA_2_α by pAKT is related to ERK^1/2^, we compared the regulation of cPLA_2_α by these two oncogenic proteins. Blocking MEK, the upstream regulator of ERK^1/2^, with MEK inhibitor (U0126) in LNCaP cells decreased pERK^1/2^ and pcPLA_2_α at Ser^505^ without changing total cPLA_2_α protein levels (Figure 4a). However, when LNCaP cells were transfected with Myr-AKT, both total cPLA_2_α and pcPLA_2_α protein levels were increased. Moreover, blocking MEK with U0126 in the Myr-AKT transfected LNCaP cells, only the pcPLA_2_α but not total cPLA_2_α protein levels were decreased (Figure 4b). Hence, the regulation of cPLA_2_α by pAKT is *via* a mechanism different from the MAP kinase ERK^1/2^.

### The decay rate of cPLA_2_α protein is dependent on pAKT levels

We notice that the magnitude of change in cPLA_2_α and pcPLA_2_α in response to pAKT is similar. Hence, pAKT may regulate cPLA_2_α expression at gene transcription level. To determine if pAKT influences cPLA_2_α expression at mRNA levels, we measured the steady-state level of cPLA_2_α mRNA by RT-qPCR. We found no change at mRNA levels of cPLA_2_α in conditions whether pAKT was increased or decreased (Supplemental Figure 2). Hence, it suggests that regulation of cPLA_2_α by pAKT occurs at post-transcriptional levels.

**Figure 5:**
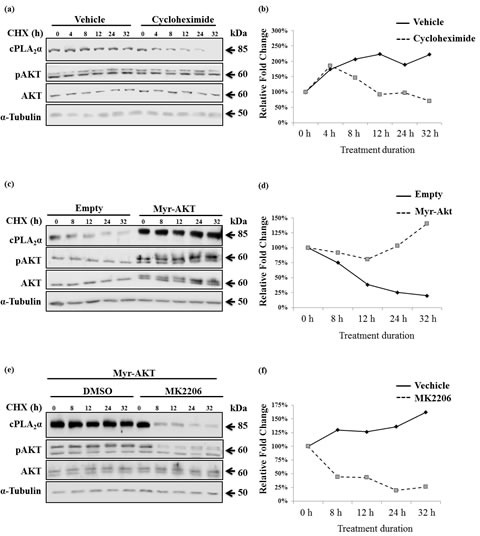
pAKT protects cPLA_2_α protein from degradation (a) PC-3 cells were treated with 50 μM cycloheximide (CHX) for the indicated time. The cells were harvested and subjected to immunoblot analysis. (b) PC-3 cells were transfected with empty vector or Myr-AKT (2 μg, 24 h) followed by incubation with CHX (50 μM) for the indicated time; and (c) PC-3 cells were transfected with Myr-AKT (2 μg, 24 h) followed by incubation with MK2206 (5 μM, 1 h). The cells were then treated with 50 μM CHX for the indicated time. All cPLA_2_α protein levels were normalized by α-tubulin. The ratio of cPLA_2_α over α-tubulin at time zero of CHX treatment was set as 100%. AKT antibodies detect AKT1 (62 kDa), AKT2 (56 kDa) and AKT3 (62 kDa, expressed mainly in the brain). When the same concentration of PAGE was used, the separation of AKT 1 from 2 is pending on the running time. The Myr-AKT1 migrates closely with the AKT1 on PAGE.

We then examined if cPLA_2_α protein stability was affected by pAKT. After determination of the decay rate of cPLA_2_α protein in the presence of cycloheximide (Figure 5a, b), PC-3 cells were transfected with Myr-AKT or empty vector followed by incubation with cycloheximide. The decay rate of cPLA_2_α protein in the presence of Myr-AKT was significantly slowed compared with empty vector-transfected cells (Figure 5c, d). To verify the necessity of pAKT in stabilizing cPLA_2_α, AKT inhibitor MK2206 was then introduced to Myr-AKT transfected PC-3 cells. Compared with vehicle-treated control cells, AKT inhibitor significantly accelerated the decay rate of cPLA_2_α protein (Figure 5e, f). It appears that the degradation of cPLA_2_α is not *via* proteasome system. Treatment with proteasome inhibitor (MG132), which clearly increased those proteins (p21 and p27) known to be degraded *via* ubiquitin-proteasome system, had little effect on cPLA_2_α (Supplement Figure 3).

**Figure 6:**
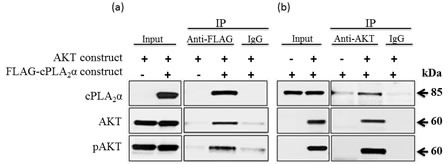
AKT (pAKT) forms a protein complex with cPLA_2_α HEK293 cells were co-transfected with (a) a Myr-AKT construct plus FLAG-cPLA_2_α construct or a FLAG-empty vector; and (b) a FLAG-cPLA_2_α construct plus a Myr-AKT construct or Myr-empty vector for 36 h. The cells were stimulated with EGF (25 ng/ml, 15 min) to increase AKT phosphorylation. Proteins were extracted and co-immunoprecipitated with a control IgG, an anti-FLAG, or an anti-AKT antibody (Ab, 2 μg each). Eluted proteins were immunoblotted with an anti-cPLA_2_α, anti-AKT or anti-pAKT Ab.

To differentiate whether AKT per se or its target proteins impinge on cPLA_2_α, we determined the effect of blocking GSK3β (the immediate downstream effector of AKT) on cPLA_2_α. If this regulation of cPLA_2_α is downstream of AKT, blocking GSK3β will result in a similar change as PTEN restoration or PI3K inhibition. Interestingly, we found no change in total and pcPLA_2_α proteins levels despite the successful inhibition of GSK3β (Supplement Figure 4). We then examined the possibility for pAKT and cPLA_2_α forming a complex. Human embryonic HEK 293 cells were transiently co-transfected with (i) a Myr-AKT construct and a FLAG-cPLA_2_α construct or a FLAG-empty vector; and (ii) a FLAG-cPLA_2_α construct and a Myr-AKT construct or a Myr-empty vector, followed by stimulation with EGF for 15 min to increase AKT phosphorylation. We then performed protein co-immunoprecipitation with an anti-FLAG or an anti-AKT antibody. Anti-FLAG antibody co-immunoprecipitated total AKT and more efficiently pAKT. Similarly, anti-AKT antibody co-immunoprecipiated cPLA_2_α (Figure 6a, b). Taken together, pAKT increases cPLA_2_α protein expression through a decrease in its protein degradation. The protection of cPLA_2_α by pAKT is through forming a complex between cPLA_2_α and pAKT.

## DISCUSSION

This study has revealed a previously-unrecognized concordant change between pAKT and lipid modifying enzyme cPLA_2_α protein levels in *PTEN*-knockout mouse prostate cancer tissues. We have also demonstrated a role of pAKT in protecting cPLA_2_α from degradation in two human prostate cancer cell lines.

LNCaP is a human lymph node metastasis-derived prostate cancer cell line with a frame-shift mutation in *PTEN* gene [29]. PC-3 is a bone metastasis-derived prostate cancer cell line with a deletion in *PTEN* gene [31]. Consequently, pAKT levels are elevated in both cell lines. By determining the response of cPLA_2_α to the decrease in pAKT, as a result of restoration of PTEN that affects mainly pAKT, we are able to ascertain the effect of a decrease in pAKT on cPLA_2_α expression and phosphorylation. Further, by blocking enzymatic action of PI3K that also affects mainly pAKT; we can mimic the decrease of pAKT independent of PTEN. It is clear from these genetic (Dox-induced or stable expression of PTEN) and pharmacological (blocking PI3K with LY204002) approaches that a decrease in pAKT leads to a decrease in total and pcPLA_2_α at Ser^505^.

To verify the finding aforementioned, we then used Myr-AKT to mimic an increase in pAKT in both LNCaP and PC-3 cells. Due to the inclusion of sequence coding for myristoylation in AKT construct, the produced AKT protein is able to bind to membrane and being phosphorylated there without using PIP3 as the membrane anchor site. We noted a significant increase in cPLA_2_α expression and phosphorylation in both cell lines following transfection of Myr-AKT. Moreover, Myr-AKT is able to override the suppressive effect of PTEN restoration on cPLA_2_α expression and phosphorylation.

As Myr-AKT increase both total AKT and pAKT, we introduced inhibitor of AKT phosphorylation in the presence or absence of Myr-AKT in both cell lines. By decreasing only the pAKT, two AKT inhibitors, AktX and MK2206, abolished the stimulatory effect of Myr-AKT on cPLA_2_α expression and phosphorylation. Together with the cPLA_2_α response to the decreased pAKT *via* restoration of PTEN or inhibition of PI3K, it is strongly suggestive that the regulation of cPLA_2_α is *via* pAKT not PTEN or PI3K.

Previous studies have demonstrated that the enzyme responsible for phosphorylation of cPLA_2_α at Ser^505^ is the phospho-ERK^1/2^ (pERK^1/2^) [34]. We have provided evidence in this study that pAKT influences cPLA_2_α including its phosphorylation at Ser^505^. To differentiate the action between pERK^1/2^ and pAKT on cPLA_2_α, we determined the effect of blocking MEK, the kinase responsible for phosphorylation of ERK^1/2^, on cPLA_2_α and pcPLA_2_α. Blocking MEK and consequently pERK^1/2^ only decreases pcPLA_2_α not total cPLA_2_α, with or without an increased AKT by Myr-AKT. Hence, the regulation of cPLA_2_α by pAKT is different from pERK^1/2^ as pAKT affects both cPLA_2_α expression and phosphorylation. Considering it is common to have an alteration in *PI3K* and *PTEN* in various cancers, it will be interesting to determine whether cPLA_2_α protein expression and phosphorylation also increase in those cancers.

Regarding the mechanism by which pAKT influences cPLA_2_α levels, as virtually in all experimental systems we adopted in the study that either increaesed or decreased pAKT, both cPLA_2_α expression and phosphorylation altered. Thus, the change in phospho-cPLA_2_α level at Ser^505^ can be at least partially a consequence of change in cPLA_2_α expression. Hence, we examined the possibility of pAKT affecting cPLA_2_α gene experssion. However, we saw no change in the steady-state levels of cPLA_2_α mRNA in all systems used in this study, including PTEN restoration and ectopic expresson of Myr-AKT. These results suggest that the effect of phospho-AKT on cPLA_2_α expression and phosphorylation is post-transcriptional. Thus, we determined the decay rate of cPLA_2_α protein following blockade of protein synthesis. The decay rate of cPLA_2_α was significantly slowed in Myr-AKT transfected cells compared with that in empty vector-transfected cells. Moreover, this slowed decay rate of cPLA_2_α protein could be reversed by inhibition of AKT phosphorylation. Hence, it is likely that pAKT promotes cPLA_2_α protein expression and phosphorylation by increasing cPLA_2_α protein stability. Moreover, we have obtained evidence based on the co-immunoprecipitation assay that pAKT and cPLA_2_α form a complex. This is consistent with our finding that blocking GSK3β has no effect on cPLA_2_α protein levels (Supplement Figure 4), making it unlikely that effectors downstream of AKT regulate cPLA_2_α. As blocking proteasome with MG132 for 24 hours does not increase cPLA_2_α protein levels (Supplement Figure 3), further study with extended time is needed to determine the mechanism by which AKT stabilizes cPLA_2_α from degradation. We searched for the AKT1 phosphorylation site based on cPLA_2_α protein sequence (PA24A_Human, UniProt # P47712) using the Group-based Prediction System (GPS) software Version 3.0 (http://gps.biocuckoo.org). Four sites were predicted to be potentially phosphorylated by AKT1: Ser^2^, Thr^321^, Thr^416^ and Ser^729^. Further study is needed to determine if these sites can be phosphorylated by AKT *in vitro* and *in vivo*, and the corresponding change in cPLA_2_α protein stability.

Interestingly, this is not the first evidence to show a regulation of a lipid modifying enzyme by AKT. AKT activation increases fatty acid synthase expression in prostate cancer tissue [35-38]. Fatty acid synthase catalyzes the conversion of acetyl-CoA and malonyl-CoA to fatty acid. Our study indicates that AKT has a similar role in regulating fatty acid cleaving enzyme cPLA_2_α in prostate cancer. Together with our recent reports on regulation of AKT by cPLA_2_α and its enzymatic products [20], these findings suggest the presence of feed-forward loop between AKT and lipid modifying enzymes that favours cancer cell proliferation. Not surprisingly, it has been suggested that chemotherapy targeting both AKT signaling and lipid metabolism might be of benefit [39, 40].

In conclusion, we have unravelled a novel regulation of cPLA_2_α expression and phosphorylation by activation of AKT. In light of the biological significance of cPLA_2_α on cell survival, proliferation, and metabolism, the role of pAKT in promoting cPLA_2_α protein stability provides a new node of interaction between oncogenic pathway and lipid metabolism and/or inflammation.

## MATERIALS AND METHODS

### PTEN-knockout mouse

The prostate epithelial-specific *PTEN*-knockout mouse was generated by Cre-loxP system. A Probasin-Cre line [41] (kindly provided by Dr. Fen Wang, The Center for Cancer Biology and Nutrition, Houston) was crossed with the PTEN^flox^ line [42]. Cre negative littermates on FBV/N genetic background were used as the wide-type control. Male mice were collected at median age of 7 weeks. Individual prostate lobes were dissected free of periprostatic fat and connective tissue and weighed. Lobes were either snap frozen with liquid nitrogen and stored in -80 °C for further protein extraction or fixed in 4% paraformaldehyde over night for histology and immunohistochemistry.

### Immunohistochemistry

The mouse prostate tissue was processed for paraffin blocks. Subsequently, paraffin sections were cut and baked at 60 °C for 1 h, after which the sections were deparaffinized, re-hydrated and subjected to antigen retrieval in Tris-EDTA solution [43]. The sections were blocked with 10% horse serum, and then incubated with an antibody to PTEN (Cat. #: 9559, Cell Signaling Technology) overnight at 4 °C. The sections were then washed with TBS and sequentially labeled with a biotinylated secondary antibody and Vectastain ABC kit (Vector Laboratories). The labeled PTEN in the sections was revealed with DAB (DakoCytomation). Thereafter, the sections were counterstained with hematoxyline and coverslipped.

### Cell culture and chemicals

The bone metastasized prostate cancer cell line, PC-3 (Cat. #: CRL-1435; American Type Culture Collection [ATCC], Manassas, VA), and the lymph node metastasized prostate cancer cell line, LNCaP (Cat. #: CRL-1740; ATCC), were grown in RPMI 1640 supplemented with 10% v/v fetal bovine serum (FBS; AusGeneX, Brisbane, QLD, Australia), penicillin (100 U/mL; Invitrogen, Melbourne, VIC, Australia) and streptomycin (100 μg/mL; Invitrogen). The cells were cultured at 37°C in an incubator providing a humidified environment in the presence of 5% CO_2_ /95% air. Incubation with various inhibitors began 24 h post seeding. LY294002 (Cat. #: S1105), MK2206 (Cat. #: S1078), U0126 (Cat. #: S1102), and SB216763 (Cat. #: S1075) were purchased from Selleck (Houston, TX). Cycloheximide (Cat. #: C1988), MG132 (Cat. #: C2211), and EGF (Cat. #: E9644) were from Sigma-Aldrich (St. Louis, MO). AktX (Cat. #: 124020) was from Calbiochem (MERCK MILLIPORE, Victoria, Australia).

### Stable and transient transfection

LNCaP carrying *tet* repressor (kindly provided by Dr. P Russell, University of NSW, Australia) were transfected with 20 μg of pcDNA4/TO (Cat. #: V1020-20; Invitrogen) vector with *PTEN* or *cPLA_2_α* sequence using Lipofectamine 2000 (Cat. #: 11668; Invitrogen). The transfected cells were selected by incubation with media containing Blasticidin (5 μg/mL, Invitrogen Cat. #: R210-01) and Geneticin Sulfate (300 μg/mL, Invitrogen Cat. #: 10131) for 10 days. Corresponding control clones were obtained by transfecting cells with the same vector without the PTEN or cPLA_2_α cDNA sequence. For induction of PTEN or cPLA_2_α, cells were seeded in 6-well plates and treated with 100 ng/mL of Doxycycline in RPMI/FBS for 24 h. PC-3 stably transfected with PTEN was a gift from Dr Zaklina Kovacevic (The University of Sydney). For transient transfection, LNCaP and PC-3 cells were transfected with 2 μg Myr-AKT (Cat. #: 9008, Addgene, Cambridge, MA) or control vector using Lipofectamine 2000. The cells were incubated for 48 h followed by RNA or protein extraction. Incubation with various inhibitors began 24 h post transfection.

### Measurement of cell proliferation and apoptosis

Apolive-Glo Multiplex assay (Cat #: G6410 and G6411) from Promega (Madison, WI) was used to determine the biological effect of PTEN restoration. Briefly the assay determines live cell using a cell membrane permeable fluorogenic peptide which is cleaved by live cell protease activity and generate a fluorescent signal. For the apoptosis, a luminogenic substrate attached to a tetramer peptide is cleaved by Caspase 3/7 activation thereby releasing a glow type luminescent signal.

### Protein Co-Immunoprecipitation

Human embryonic HEK 293 cells were transiently transfected with pCMV1-Flag-cPLA_2_α, pcDNA-Myr-AKT or both with Lipofectamine2000 (Invitrogen) for 36 h. Total protein (0.5 mg) was incubated overnight with 2 μg of anti-Flag, anti-AKT or control IgG antibody. Eluted proteins were immunoblotted with anti-cPLA_2_α, anti-AKT, and anti-pAKT antibodies. Refer to previous publication for details of methodology [44].

### Immunoblotting

PC-3 and LNCaP cells were treated in 6-well plates, and cell lysates were prepared in a lysis buffer as described previously [23]. To detect proteins of interest in mouse prostate tissues, previously frozen tissues were weighed out and added with 20-fold volume of the lysis buffer and homogenized in 1.5-mL Eppendorf tubes on ice. The homogenates were then centrifuged at 12,000 g for 1 min and resultant supernatants were collected and stored at -80 °C until use. Two loading controls, α-tubulin and glyceraldehyde 3-phosphate dehydrogenase (GAPDH), were used to avoid overlapping signals from proteins of interest which had a similar molecular weight. Primary antibodies against: cPLA_2_α (Cat. #: SC-454), phospho-cPLA_2_α at Ser^505^ (Cat. #: SC-34391), AKT (Cat. #: SC-8312), and phospho-AKT at Ser^473^ (Cat. #: SC-7985) were purchased from Santa Cruz Biotechnology (Santa Cruz, CA); ERK 1/2 (Cat. #: 9102), phospho-ERK 1/2 (Cat. #: 9106S), GSK3β (Cat. #: 9315) and phospho-GSK3β (Cat. #: 9336S) were bought from Cell Signaling Technology (Danvers, MA); Anti-FLAG-Ab (Cat. #: F3165) were purchased from Sigma; α-tubulin (Cat. #: ab7291) and GAPDH (Cat. #: ab8245) were obtained from Abcam (Boston, MA).

### Reverse Transcription-and quantitative real-time PCR

Total RNA was isolated using the UltraClean Tissue & Cells RNA Isolation Kit (Mo Bio Laboratories, CA). The first strand cDNA was synthesized from 500 ng of total RNA with random hexamers and SuperscriptIII (Cat. #: 48190 and P/N 56575, Invitrogen). The primers used for cPLA_2_α were forward 5'-ATCCTGATGAATTTGAGCGA and reverse 5'CAAGTAGAAGTTCCTTGAACG. The TATA box binding protein (TBP) was used as the housekeeper gene, forward 5'-GAACCACGGCACTGATTTTC, reverse 5'-CCCCACCATGTTCTGAATCT. Quantitative PCR measurements were performed using a SensiMix SYBR Mastermix Kit (Cat. #: QT605, Bioline, Sydney, NSW, Australia) and a RotorGene 6000 PCR machine (Qiagen, Santa Clarita, CA). Conditions for PCR were one cycle of 10 min at 95 °C; 40 cycles of 10 s at 95 °C and 30 s at 60 °C. The Relative Expression Software Tool 2009 (Qiagen) was used to calculate relative changes in cPLA_2_α normalized to the housekeeping gene. Amplification efficiency was determined using a 5-point dilution curve and was within 100% ± 3% for cPLA_2_α and TBP.

### TCF/LEF promoter assay

The dual luciferase activity of TCF/LEF (TOPO-Flash) promoter assay (Cat. #: E1910) from Promega was used to verify the inhibition of GSK3β by measuring β-catenin activity.

### Statistical Analysis

The statistical software NCSS (v12.0; Kaysville, UT) was used for analysis. One-Way ANOVA was implemented to determine the difference between individual groups of data. Fisher's LSD Multiple-Comparison Test was used to determine whether the difference between individual groups (*P*<0.05) was considered significant.

## SUPPLIMENTARY MATERIAL AND FIGURES


