# Supplementary material for: Loss of PTEN stabilizes the lipid modifying enzyme cytosolic phospholipase A2α via AKT in prostate cancer cells
Source: Oncotarget. 2014 Jul 9;5(15):6289–99. doi: 10.18632/oncotarget.2198 (PMC4171630; doi:10.18632/oncotarget.2198)
Supplement: Supplementary file 1 [file oncotarget-05-6289-s001.pdf]

Loss of PTEN stabilizes the lipid modifying enzyme cytosolic phospholipase A2α *via* AKT in prostate cancer cells

Supplimentary Material

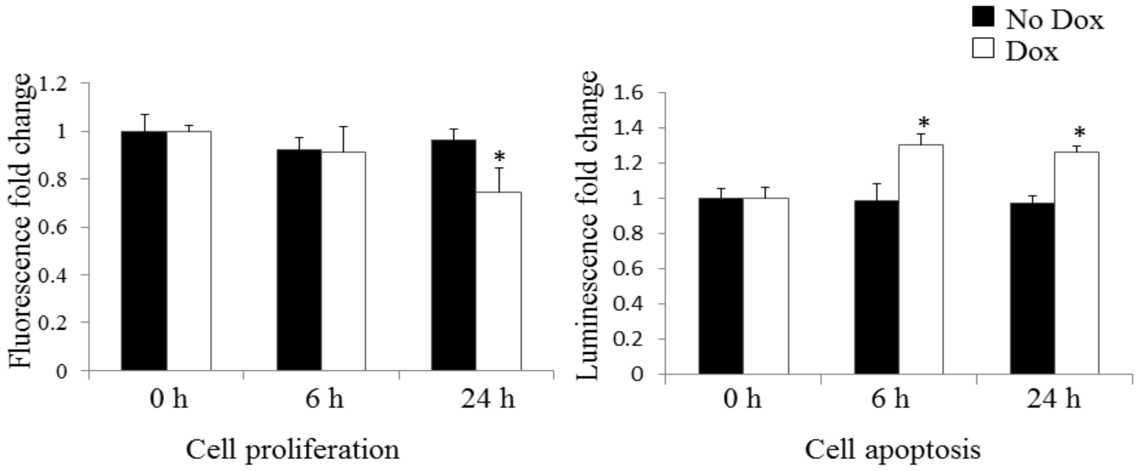

Figure S1: LNCaP cells transfected with Dox-controlled inducible PTEN expression system were treated with or without Dox (100 ng/mL) for 24 h. Apolive-Glo Multiplex assays (Promega) were used to determine the effect of PTEN expression on cell proliferation (a) and apoptosis (b). Results represented as the mean ± SD. * $P<0.05$ versus no Dox treatment.

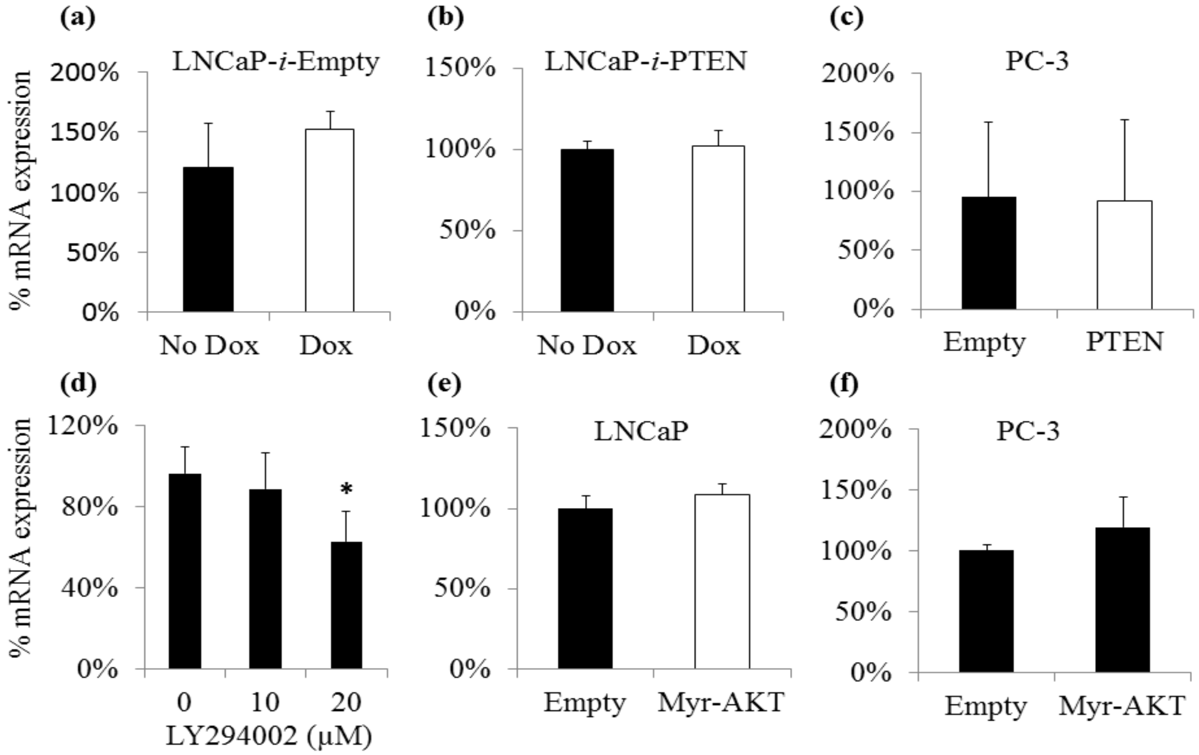

Figure S2: Restoration of PTEN expression, inhibition of PI3K, or over-expression of Myr-AKT on cPLA$_2$α mRNA levels by RT-qPCR. The experimental cells as described for immunoblotting were harvested. Total RNA was extracted followed by reverse transcription-and quantitative real-time PCR as described under Methods. The Relative Expression Software Tool 2009 (Qiagen) was used to calculate relative changes in cPLA$_2$α normalized to the housekeeping gene (TBP). Amplification efficiency was determined using a 5-point dilution curve and was within 100% ± 3% for cPLA$_2$α and TBP. * $P<0.05$ versus control.

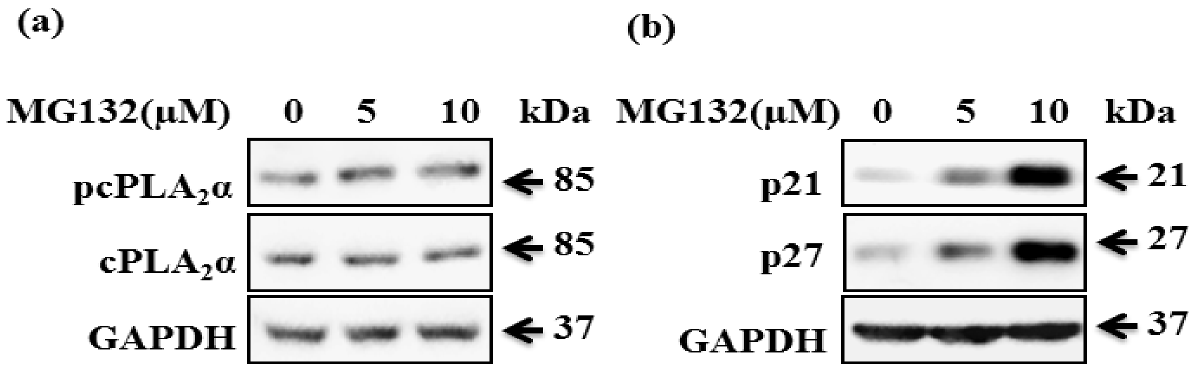

Figure S3: Effect of protease inhibitor MG132 on cPLA$_2$α protein levels. PC-3 cells were treated with the indicated dose of MG132 for 24 h. The cells were harvested and subjected to immunoblot analysis. MG132 had little effect on cPLA$_2$α (a) but clearly increased p21 and p27(b).

(a)

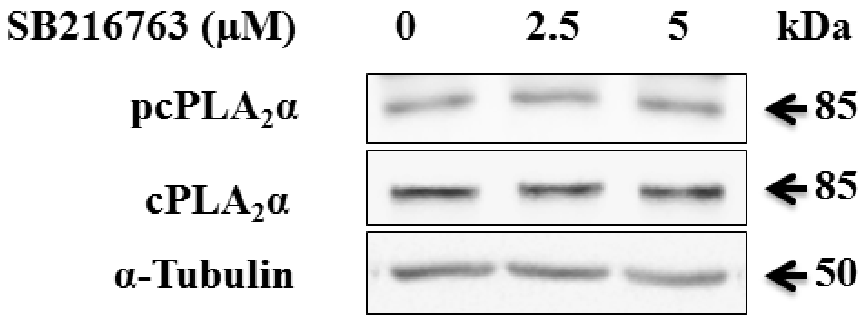

(b)

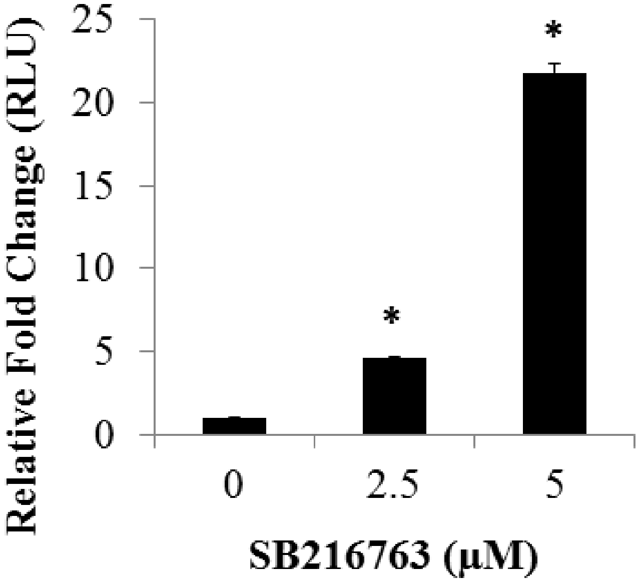

Figure S4: The effect of blocking GSK3β (the immediate downstream effector of AKT) on $cPLA_2\alpha$. To differentiate whether AKT per se or its target proteins impinge on $cPLA_2\alpha$, PC-3 cells treated with the indicated dose of GSK3β inhibitor for 24 h and subjected to immunoblot (a). Total and $pcPLA_2\alpha$ proteins levels were unchanged despite the successful inhibition of GSK3β as shown by the dual luciferase activity of TCF/LEF (TOPO-Flash) promoter assay measuring β-catenin activity (b).
